# New Insights into Asian Prunus Viruses in the Light of NGS-Based Full Genome Sequencing

**DOI:** 10.1371/journal.pone.0146420

**Published:** 2016-01-07

**Authors:** Armelle Marais, Chantal Faure, Thierry Candresse

**Affiliations:** 1 INRA, UMR 1332 BFP, Villenave d’Ornon, France; 2 Université de Bordeaux, UMR 1332 BFP, Villenave d’Ornon, France; Oklahoma State University, UNITED STATES

## Abstract

Double stranded RNAs were purified from five *Prunus* sources of Asian origin and submitted to 454 pyrosequencing after a random, whole genome amplification. Four complete genomes of Asian prunus virus 1 (APV1), APV2 and APV3 were reconstructed from the sequencing reads, as well as four additional, near-complete genome sequences. Phylogenetic analyses confirmed the close relationships of these three viruses and the taxonomical position previously proposed for APV1, the only APV so far completely sequenced. The genetic distances in the respective polymerase and coat protein genes as well as their gene products suggest that APV2 should be considered as a distinct viral species in the genus *Foveavirus*, even if the amino acid identity levels in the polymerase are very close to the species demarcation criteria for the family *Betaflexiviridae*. However, the situation is more complex for APV1 and APV3, for which opposite conclusions are obtained depending on the gene (polymerase or coat protein) analyzed. Phylogenetic and recombination analyses suggest that recombination events may have been involved in the evolution of APV. Moreover, genome comparisons show that the unusually long 3’ non-coding region (3' NCR) is highly variable and a hot spot for indel polymorphisms. In particular, two APV3 variants differing only in their 3’ NCR were identified in a single *Prunus* source, with 3' NCRs of 214–312 nt, a size similar to that observed in other foveaviruses, but 567–850 nt smaller than in other APV3 isolates. Overall, this study provides critical genome information of these viruses, frequently associated with *Prunus* materials, even though their precise role as pathogens remains to be elucidated.

## Introduction

The Asian prunus viruses (APV) were initially identified in several *Prunus* sources of Asian origin showing cross-reactivity to *Plum pox virus* (PPV), the viral agent causing Sharka disease, the most important virus disease on stone fruit trees [1, 2]. They were therefore initially diversely called "Plum pox-like virus", "*Prunus* latent virus" or "*Prunus* virus isolates" [2–4]. Several polyclonal antisera showed reliably a cross-reactivity with APV *Prunus* sources, whereas PPV-specific monoclonal antibodies failed to react [2]. There are some indications that APV are not responsible for the PPV cross-reactivity, because some of the original PPV-reacting *Prunus* sources were not found to be infected by APV and since the *in vitro* translated coat protein of APV failed to react with a PPV antiserum [5]. APV have also been shown to cross-react in immunosorbent electron microscopy and Western blot analyses with polyclonal antisera directed against *Apple stem pitting virus* (ASPV) [4], the type member of the genus *Foveavirus* in the family *Betaflexiviridae*.

An initial molecular characterization suggested the existence of three distinct but related agents which were accordingly named Asian prunus virus 1, 2 and 3 [5, 6]. The first complete genome of Asian prunus virus 1 (APV1) was reported by Marini et al. [7]. The partial characterization of the related Asian prunus virus 2 (APV2) and Asian prunus virus 3 (APV3) led to the suggestion that they belonged, together with APV1, to the genus *Foveavirus* in the family *Betaflexiviridae* [5]. However, the lack of complete genome sequences for APV2 and APV3 has thus far hampered the clarification of the taxonomic position of APV, in particular whether the three agents should be considered as a single or as three distinct viral species.

The study of these viruses has been complicated by the woody nature of the host plants and by the fact that some of the *Prunus* sources showing the cross-reactions with ASPV and PPV are frequently also co-infected with well-known fruit tree viruses and viroids such as *Apple chlorotic leafspot virus* (ACLSV) and *Peach latent mosaic viroid* (PLMVd) [8, 9]. In this context, the developments of high-throughput sequencing technologies and of bioinformatic tools to efficiently manipulate the large amount of sequence data thus generated, have drastically impacted the plant virus detection and characterization field [10–12]. This is reflected in the rapid increase in the number of novel plant RNA and DNA viruses in the past five years [13], and in our better understanding of the molecular diversity of known viruses, allowing the development of detection assays of improved broad-spectrum and specificity [14].

In order to clarify the taxonomical position of APV1, 2 and 3, and the virological status of the PPV-reacting *Prunus* sources, double-stranded RNAs (dsRNAs) from five *Prunus* of Asian origin known to cross-react with PPV were purified and submitted to pyrosequencing. The results obtained allowed assembly of several complete or near complete genomic sequences of APV1, 2, and of 3 and indicate that APV2 represents a distinct species in the genus *Foveavirus* while the precise status of APV1 and APV3 is more complex to establish.

## Materials and Methods

### Plant samples and virus isolates

The Japanese apricot (*Prunus mume*) cv. Bungo (Q1256-01) and the peaches (*Prunus persica*) cv. Ta Tao 23 (Q-375-23), and cv. Ta Tao 25 (Q-375-02) were provided by Dr. A. Hadidi (USDA Beltsville, USA) and are among the six original PPV cross-reacting *Prunus* sources reported by James et al. (1994). The Bonsai source was obtained from Dr. J.B. Quiot (INRA Montpellier, France). This tree is a *P*. *mume* bonsai from Japan intercepted in 1993 by the French Plant Protection Service because this tree tested positive for PPV. The peach (*P*. *persica*) Nanjing source was collected during survey in China in 2009 [14]. All sources were propagated by grafting on GF305 peach seedlings under BL3 containment greenhouse conditions.

### Double-stranded RNAs extraction and pyrosequencing

Double-stranded RNAs (dsRNAs) were extracted from fresh leaves of GF305 peach grafted with each of the APV sources following the protocol described by Gentit et al. [15] with the modification introduced by Candresse et al. [16]. After a random reverse transcription step and a whole-genome amplification, the fragments were analyzed by 454 pyrosequencing, following the strategy reported by Candresse et al. [16]. After demultiplexing, the reads were assembled using the CLC Genomics Workbench 7.0 (http://www.clcbio.com) and annotated by BlastX and BlastN comparison with GenBank, using a 10^−3^ e-value cut-off. The scaffolding and ordering of the contigs for each viral isolate were facilitated by mapping the contigs on reference viral genomes. The gaps between the contigs as well as regions of low pyrosequencing coverage were amplified from total nucleic acids (TNA, [9]), extracted from the grafted GF305 leaves, using primers designed from the sequence of the contigs (S1 Table) in a two-step RT-PCR procedure described by Marais et al. [17]. 5' and 3' ends of the viral genomes were determined using either a 5' Random Amplification of cDNA Ends (5' RACE) strategy, or a Smart^™^ Long Distance-RT-PCR (Takara Bio Europe/Clontech, Saint-Germain-en-Laye, France) for the 3’ genomic regions, using internal primers designed from the assembled contigs (S1 Table). The RACE reactions were performed following the kit manufacturer's instructions (Takara Bio Europe/Clontech, Saint-Germain-en-Laye, France) and the 3’ genome ends were amplified using the protocol described by Youssef et al. [18]. All amplification products were sequenced on both strands (GATC Biotech AG, Mulhouse, France), either directly or after a cloning step into the pGEM-T Easy vector (Promega, Charbonnières-Les Bains, France). The sequences obtained were finally assembled with the 454 contigs to generate the complete genomic sequence of the virus isolates.

### Sequence and phylogenetic analyses

Analysis of 454 pyrosequencing sequence data was performed as described by Candresse et al. [16] using the CLC Genomics Workbench 7.0. Multiple alignments of nucleotide or amino acid sequences were performed using the ClustalW program as implemented in MEGA version 6.0 [19]. Phylogenetic trees were reconstructed using the neighbor-joining technique with strict nucleotide or amino acid distances and randomized bootstrapping for the evaluation of branching validity. Genetic distances (p-distances calculated on nucleotide or amino acid identity) were calculated using MEGA version 6.0. The RDP4 program [20] was used to search for potential recombination events in the APV genomic sequences obtained in this study.

## Results

### Pyrosequencing of dsRNAs extracted from the five APV sources

All sources were found to be infected with more than one virus with the exception of Bonsai. Whereas APV2 was the sole virus detected in Bonsai source, representing 77.6% of the total reads, six different viruses were found in the Ta Tao 25 source: APV2 (46.8% of the total reads), APV3 (11.7% of reads), APV1 (2.6% of reads) and three well known fruit tree viruses, *Plum bark necrosis stem pitting-associated virus* (PBNSPaV, 14.7% of reads), *Cherry green ring mottle virus* (CGRMV, 0.1% of reads), and *Apple chlorotic leaf spot virus* (ACLSV, 0.08% of reads). In the Ta Tao 23 source, Blast analysis identified contigs belonging to each of the three APV: APV1 (3% of reads), APV2 (1.5%), APV3 (26.1%), while 1.3% of the total reads corresponded to ACLSV sequences. A mixed infection with two APV was also observed in the Bungo source, involving APV2 (66.4% of total reads) and APV1 (13.1%). Finally, as shown in a previous work [14], analysis of the contigs from the Nanjing source showed the presence of APV3 (6.7% of the reads), PPV (52%) and PBNSPaV (34.3%). Further analyses of the low levels of reads observed for CGRMV or ACLSV in some of the samples showed that, in each case, the contigs covered a significant proportion of the viral genome (36 to 69%, not shown), suggesting that these viruses were really present in the samples and that the low level of reads observed did not result from a contamination.

For each source, contigs annotated as belonging to the various APV were further manually assembled into scaffolds using the APV1 genome [7] as a reference. The partial genome sequences of APV2 and APV3 [5] were also used as references in this scaffold assembly process. The scaffolds were then further extended using a combination of reads mapping and de novo assembly [16]. From the scaffolds thus obtained, four were selected for completion of the sequence of the corresponding isolate: the APV1 from the Bungo source (four internal gaps and 5' and 3' ends missing), the APV2 isolates from the Bungo and Bonsai sources (both missing one short internal region and both genome ends), and the APV3 isolate from the Nanjing source (two internal gaps and both genome ends missing). These four genomic sequences were completed by direct sequencing of RT-PCR products obtained using total nucleic acids of the respective APV sources and specific primers targeting the remaining gaps (S1 Table). The 5’ and 3’ genome ends were obtained using 5’RACE and Smart^™^ Long Distance-RT-PCR [18], respectively. The completed sequences have been deposited under accession numbers KT893293-KT893296 in the GenBank database.

In addition, the genome sequences of an additional APV2 isolate (Ta Tao 25 source) and of three additional APV3 isolates (two from the Ta Tao 23 source and one from the Ta Tao 25 source) were also obtained during the assembly process. Their 3’ genome end was completed as described above but no specific effort was made to complete the 5’ genome end, thus, depending on the isolate, between 395 to 745 nucleotides were missing. These sequences have been deposited under accession numbers KT893297 to KT893300 in the GenBank database.

### Genome organization of APV1, 2, and 3

**Fig 1:**
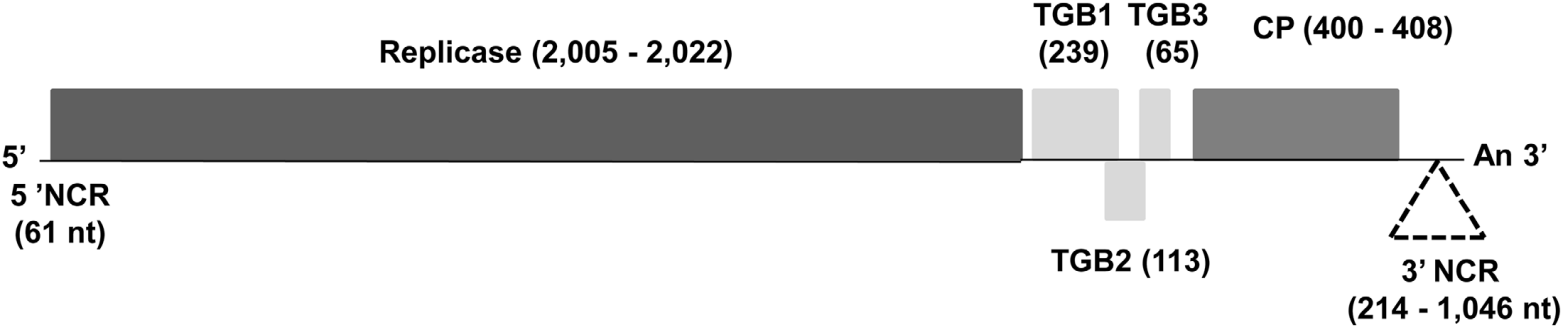
Genome organization of Asian prunus virus 1, 2, and 3. Open reading frames are represented by shaded boxes. The size ranges of the deduced proteins and of the 5' and 3' non coding regions (NCR) are indicated in amino acids and nucleotides, respectively. The dashed triangle represents the large indel region in the 3’ non-coding region. TGB1, 2, and 3: triple gene block protein 1, 2, and 3, respectively. CP: coat protein.

With the present results, complete genome sequences of two APV1 isolates (including that published by Marini et al [7], FJ824737), two APV2 isolates, and one APV3 isolate are now available. Moreover, near complete sequences, missing only 0.3 to 0.7 kb of 5’-terminal sequence, were also determined for one additional APV2 isolate and three APV3 isolates. Taken together, these sequences show that the genome organizations of APV1, APV2 and APV3 are closely similar to that described for the APV1 reference isolate [7] and are typical of members of the genus *Foveavirus* (Fig 1). The genome encodes five open reading frames (ORFs), encoding from 5’ to 3’ the polymerase, the triple gene block proteins (TGB1, 2, and 3) involved in viral movement and finally the coat protein (CP).

The genomes of the APV1 to 3 and their isolates are largely colinear. The length of the genome of APV1 Bungo (9,473 nt) is in the same range as that of the reference APV1 isolate (9,409 nt, [7]), the size polymorphism being exclusively limited to the 3' NCR, the other genomic regions being strictly colinear between the two isolates (Table 1). The genome sizes of the APV2 Bungo and Bonsai isolates are very similar (9,362 and 9,375 nt, respectively) with two regions polymorphic: the 3' NCR and the polymerase gene which displays a 39-nt long (13 amino acids) deletion in the Bungo isolate. At 9,654 nt, the APV3 Nanjing isolate has the longest genome. The sizes of the 5' NCR, the polymerase gene and TGB genes are similar to those of APV1 and APV2. The CP is slightly larger (408 aa as compared to 400 in APV1 and APV2), but the largest difference was once again in the 3’ NCR (1,046 nt), which is 160–258 nt longer than those of APV1 and APV2 (Table 1). This long 3’ NCR had previously been identified as a salient discriminating feature of APV [5] as compared to other members of the genus *Foveavirus*, in which this region is much shorter. No additional ORF was identified in this long 3' NCR.

**Table 1:** Size of the genome, 5' and 3' non coding regions and open reading frames encoded by the genome of Asian prunus virus 1, 2, and 3.

	Genome size (nt)	5' NCR (nt)	Polymerase (aa)	TGB1 (aa)	TGB2 (aa)	TGB3 (aa)	CP (aa)	3' NCR (nt)
**APV1 Ta Tao 5 Reference isolate**	9,409	61	2,022	239	113	65	401	822
**APV1 Bungo**	9,473	61	2,022	239	113	65	401	886
**APV2 Bungo**	9,362	61	2,005	239	113	65	400	814
**APV2 Bonsai**	9,375	61	2,018	239	113	65	400	788
**APV2 Ta Tao 25**	na^1^	na	na	239	113	65	400	815
**APV3 Nanjing**	9,654	61	2,022	239	113	65	408	1,046
**APV3 Ta Tao 25**	na	na	na	239	113	65	408	879
**APV3 Ta Tao 23 variant 1**	na	na	na	239	113	65	408	214
**APV3 Ta Tao 23 variant 2**	na	na	na	239	113	65	408	312

^1:^ not available

Interestingly, the 3' NCR of APV3 appears to be highly polymorphic in size among the four APV3 isolates sequenced in the present work (S1 Fig). The Ta Tao 25 APV3 isolate has a 3’ NCR of 879 nt (Table 1), a size comparable to that observed in APV1 and APV2 isolates. The difference in 3’ NCR size is mostly explained by a large, ca. 200 nt indel polymorphism (S1 Fig). In addition, in the Ta Tao 23 source, two APV3 variants differing only in their 3’ NCR were identified. These variants showed 3’ NCRs with large internal deletions, resulting in an overall length of 312 or 214 nt, a size similar to the 176–312 nt long 3’ NCRs reported for other Foveaviruses [21]. The last ca. 150 nt of the 3’ NCR were highly conserved among all APV isolates (S1 Fig).

### Phylogenetic relationships of APV1, 2, and 3

**Fig 2:**
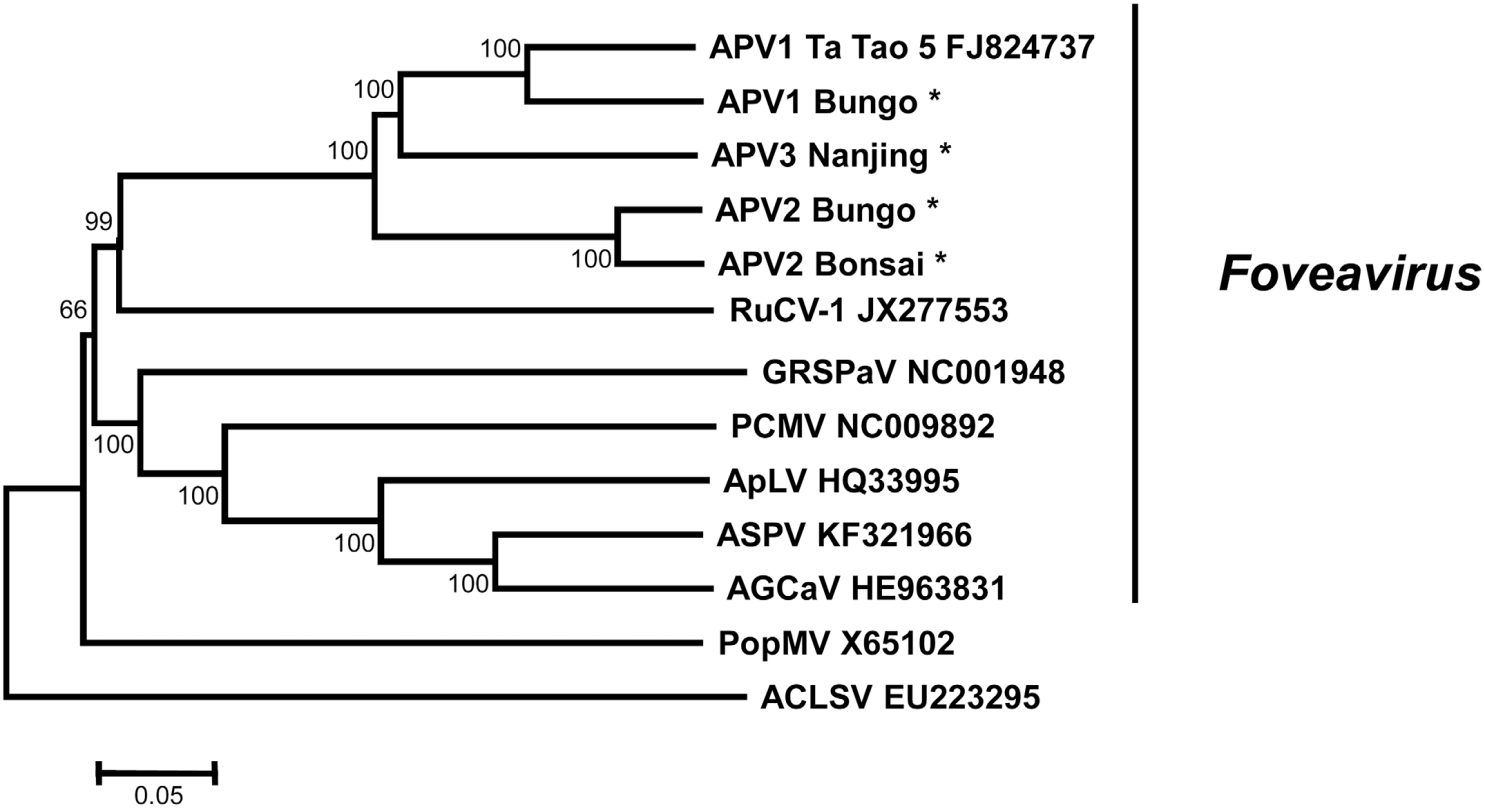
Unrooted phylogenetic tree reconstructed with the complete genome sequence of representative members of the family *Betaflexiviridae*. Tree was constructed by the neighbor-joining method and the statistical significance of branches was evaluated by bootstrap analysis (1,000 replicates). Only bootstrap values above 65% are indicated. The scale bar represents 5% nucleotide divergence. The abbreviations followed by the accession numbers are: APV1, 2 or 3, Asian prunus virus 1, 2 or 3, respectively; RuCV-1, *Rubus canadensis virus 1*; GRSPaV, *Grapevine rupestris stem pitting associated virus*; PCMV, *Peach chlorotic mottle virus*; ApLV, *Apricot latent virus*; ASPV, *Apple stem pitting virus*; AGCaV, *Apple green crinkle associated virus*; PopMV, *Poplar mosaic virus*; ACLSV, *Apple chlorotic leaf spot virus*. The sequences of APV1, 2, and 3 determined in this work are identified by an asterisk *.

Besides their similarities in genome organization, the close relationships linking APV and Foveaviruses are illustrated by a phylogenetic analysis performed on their complete genome sequences, with *Poplar mosaic virus* (PopMV, *Carlavirus*) and *Apple chlorotic leaf spot virus* (ACLSV, *Trichovirus*) being used as representatives of other *Betaflexiviridae* genera. The phylogenetic neighbor-joining tree, reconstructed using strict nucleotide sequence identity distances (Fig 2) shows that APV cluster with high bootstrap support (99%) with *Rubus canadensis virus 1* (RuCV-1), a tentative member of the genus *Foveavirus*, as well as the other *Foveavirus* members (66% bootstrap value; *Grapevine rupestris stem pitting associated virus*, GRSPaV */ Apple stem pitting virus*, ASPV / *Peach chlorotic mottle virus*, PCMV / *Apricot latent virus*, ApLV / *Apple green crinkle associated virus*, AGCaV). The average pairwise nucleotide divergence value among the five APV sequences was 23.5±0.3% and isolates of each virus clustered together. However, APV3 appears closer to APV1 and formed a bootstrap-supported cluster with the APV1 isolates (Fig 2).

In order to clarify the taxonomical status of APV in the family *Betaflexiviridae*, sequence comparisons were performed for the polymerase and coat protein genes and for the corresponding proteins (Table 2). The accepted species demarcation molecular criteria for the family *Betaflexiviridae* are of 28% nucleotide divergence or 20% amino acid divergence in the polymerase and coat protein genes [21]. By almost all criteria, APV2 appears to be a distinct species, with the exception of its polymerase amino acid divergence level which is sometimes below the 20% threshold when comparing with some APV1 or APV2 isolates. The situation is less clear for APV1 and APV3. Considering the polymerase gene, these agents show divergence values within the species variation range, irrespective of whether the nucleotide or amino acid sequences are considered (Table 2). However, when the comparisons are performed with the coat protein gene, the situation is reversed, with nucleotide divergence levels of 30 to 32.3% (25.7 to 26.9% amino acid divergence), which are significantly above the species cut-off values.

**Table 2:** Percentage of divergence in the polymerase and coat protein genes and deduced proteins between Asian prunus virus 1, 2, and 3 isolates.

	Polymerase		Coat Protein	
	Nucleotide	Amino acid	Nucleotide	Amino acid
**APV1-APV3**	25.7–27.3%	14.3–15.6%	30–32.3%	25.7–26.9%
**APV1-APV2**	28.7–29.8%	19.9–20.9%	35.5–37.5%	35–36.8%
**APV2-APV3**	28.1–29.3%	18.7–20.2%	37.3–38.8%	34.5–37%

**Fig 3:**
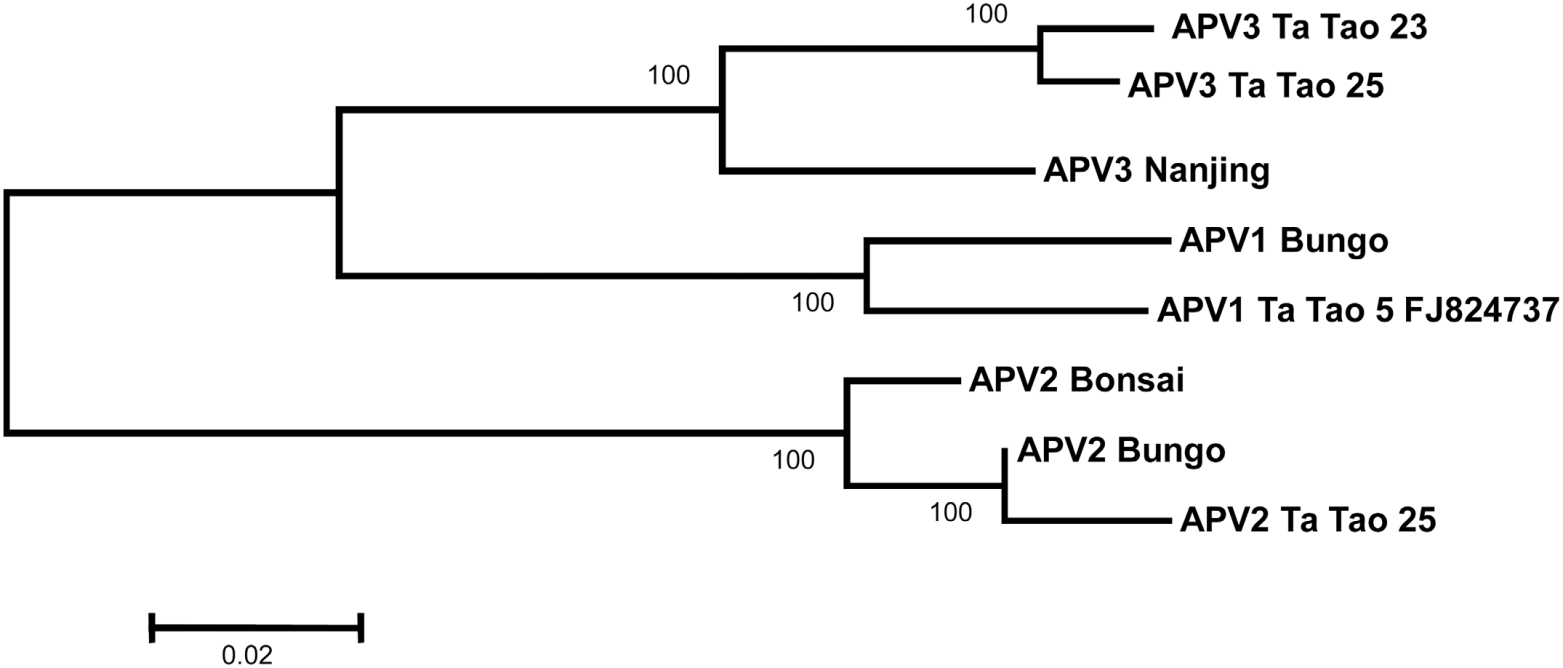
Unrooted phylogenetic tree reconstructed using the amino acid sequences from the polymerase of Asian prunus virus 1, 2, and 3 isolates. The partial polymerase sequences of APV2 Ta Tao 25, APV3 Ta Tao 23, and APV3 Ta Tao 25 were used (112, 228, or 227 amino acids missing at the NH_2_ terminal end, respectively). Tree was constructed by the neighbor-joining method and the statistical significance of branches was evaluated by bootstrap analysis (1,000 replicates). Only bootstrap values above 70% are indicated. The scale bar represents 2% amino acid divergence.

**Fig 4:**
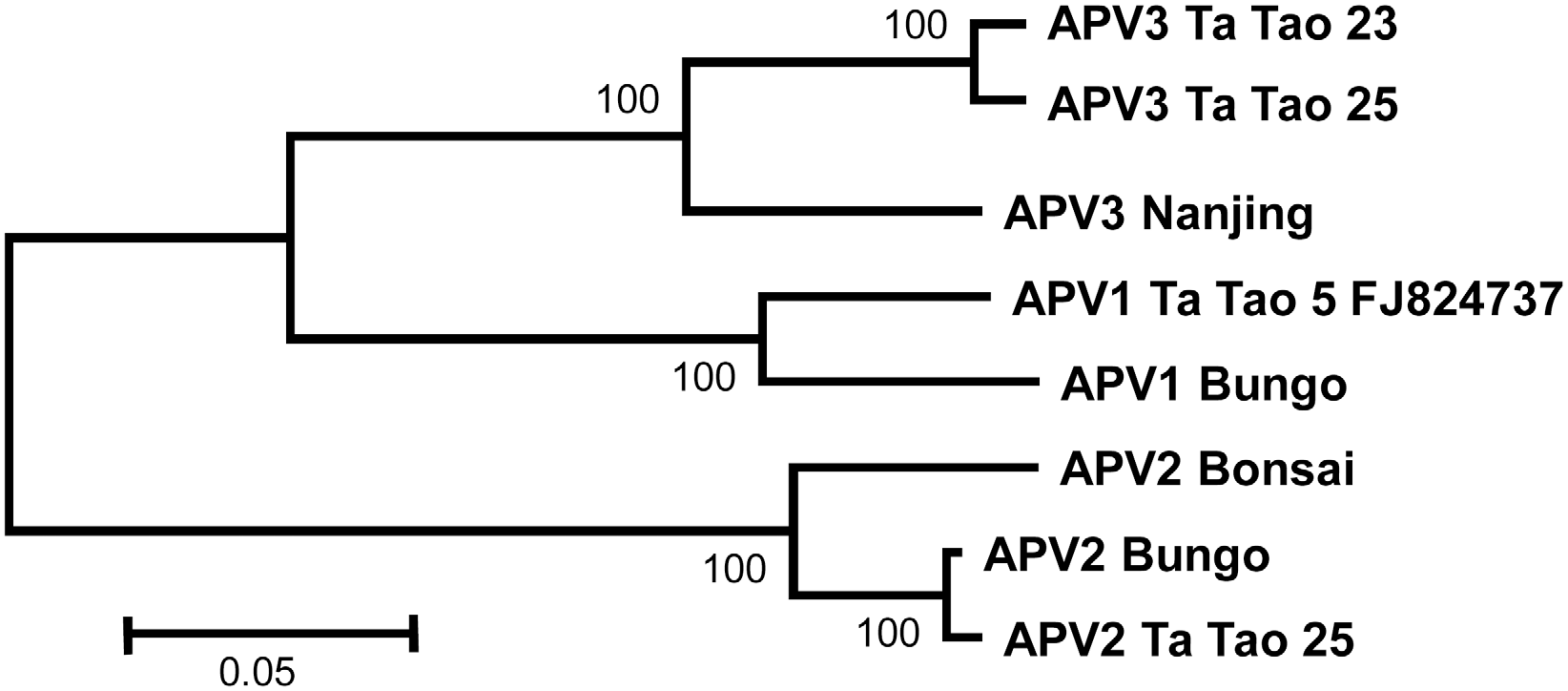
Unrooted phylogenetic tree reconstructed using the amino acid sequences from the coat protein of Asian prunus virus 1, 2, and 3 isolates. Tree was constructed by the neighbor-joining method and the statistical significance of branches was evaluated by bootstrap analysis (1,000 replicates). Only bootstrap values above 70% are indicated. The scale bar represents 5% amino acid divergence.

To complete these analyses, neighbor-joining trees were reconstructed using the coat protein and polymerase amino acid sequences (Figs 3 and 4). For the polymerase, the near complete sequences of the Ta Tao 25 APV2 isolate and of the Ta Tao 23 and Ta Tao 25 APV3 isolates were included in the analysis. The topology of both trees is similar to that of the tree reconstructed with the complete genome sequences (Fig 2) and the isolates of each agent form a distinct, 100% bootstrap-supported cluster. The close relationship linking APV1 and APV3 is also evident in both trees.

**Fig 5:**
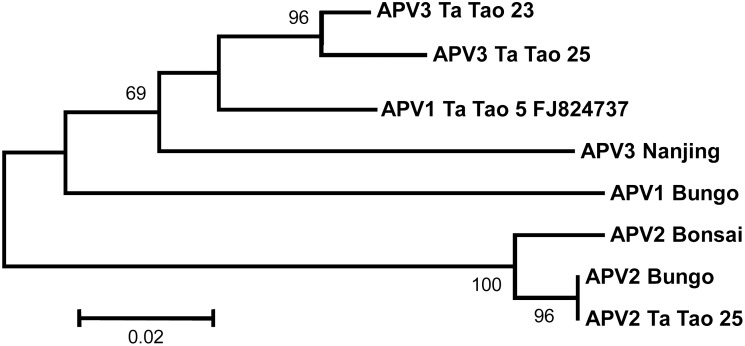
Unrooted phylogenetic tree reconstructed with the concatenated amino acid sequences of the Triple Gene Block proteins 2 and 3 of Asian prunus virus 1, 2, and 3 isolates. Tree was constructed by the neighbor-joining method and the statistical significance of branches was evaluated by bootstrap analysis (1,000 replicates). Only bootstrap values above 60% are indicated. The scale bar represents 2% amino acid divergence.

Whereas the same tree topology was again obtained when analyzing the TGB1 protein (data not shown), a different pattern emerged with the tree reconstructed using the concatenated TGB2 and TGB3 protein sequences (Fig 5). Indeed, the APV1 Ta Tao 5 reference isolate now clusters together with the APV3 isolates, but away from the other analyzed APV1 Bungo isolate. Such incongruence might be explained by a recombination event, whose potential occurrence was further evaluated using the RDP4 program. A single recombination event involving APV3 Ta Tao 25 and APV1 Ta Tao 5 was detected with very good probability (10^−14^ to 10^−44^ depending on the algorithm used). The predicted recombined fragment is approximately 500 nt long, with borders around nucleotide positions 6680 and 7189 in the Ta Tao 5 APV1 genome, corresponding to the region comprised between the end of the TGB1 and the end of the TGB2 genes.

## Discussion

The NGS strategy used here allowed the efficient determination of complete genome sequences of four APV1, 2 or 3 isolates. In addition, near complete genome sequences were also obtained for one additional APV2, and three additional APV3 isolates, confirming the potential of NGS technologies to detect and characterize fruit tree viruses, even in situations of multiple infections, like in the case of Ta Tao 25 source, where six different viruses were detected.

When compared with other foveaviruses, the eight APV isolates characterized in the present work show more than the 45% nucleotide identity, in their polymerase and coat protein genes, currently accepted genus demarcation criteria in the family *Betaflexiviridae* (data not shown). This finding supports the previous suggestion [5, 7] that APVs should be regarded as species of the genus *Foveavirus*. This conclusion is further supported by the similarities in genome organization and by the whole genome phylogenetic analysis reported here. When it comes to the species status of the various APV, the situation is more complex. Taking into account sequence comparisons between APV1, 2, and 3 in the two taxonomically relevant regions, we propose that APV2 should be considered as a distinct species in the genus *Foveavirus*, even if the amino acid identity levels in the polymerase are very close to the species demarcation criteria accepted in the family *Betaflexiviridae*. The situation of APV1 and APV3 is more complex, since sequence comparisons using the polymerase and coat protein genes or their deduced amino acid sequences provide a conflicting picture, with divergence levels suggesting the existence of a single or of two species, respectively. Although molecular criteria based on identity level in the polymerase and in the coat protein genes are usually convergent [21], such a situation of conflict between polymerase and coat protein criteria has been observed previously for a few Foveaviruses [18, 22, 23] or for unassigned members in the family *Betaflexiviridae* [24]. In such cases, the use of additional biological information such as serology, host range, associated symptoms, or vector transmission has been used to reach a decision on the species status. There is a need for such additional information to determine if APV1 and APV3 constitute a single or two distinct species.

Conflicts between polymerase and coat protein identity levels used as taxonomic criteria seem to be particularly frequent in the genus *Foveavirus* ([18, 22, 23], present work). This situation appears to be a consequence of the particularly long hypervariable N-terminal region of the coat protein in this genus, which results in high divergence values between isolates and species even if the C-terminal part of the coat protein is highly conserved [18]. Since this appears to be a peculiarity of the genus *Foveavirus* within the family *Betaflexiviridae*, a revision of the species discrimination criteria in this genus to take into account may be required ultimately.

Phylogenetic analyses on various APV species performed using the amino acid sequences of the various APV proteins revealed that the TGB2-TGB3 tree was not congruent with the trees generated using other proteins (compare Figs 3–4 and 5). This observation as well as RDP4 analysis strongly suggest that the APV1 Ta Tao5 reference isolate is in fact an APV1-APV3 recombinant in the TGB region. Previous studies have shown that recombination is a relatively common process in RNA plant virus evolution [25, 26], even the rate of recombination differs across virus genera. Indeed, recombination events have previously been reported to be involved in the evolution of some *Betaflexiviridae* members [27–32], and additional cases are likely to be documented in the future as more genus members are characterized through metagenomic studies [33].

Recombination events are similarly the most likely explanation for the very large indel polymorphisms observed in the 3’ NCRs of APV3 isolates. The identification of APV3 isolates with small, 214 or 312 nt-long 3’ NCRs is interesting in that it shows that functional APV genomes can exist with 3’ NCRs of a size similar to those of other genus members. In the absence of any additional coding potential, the selective advantage that might be conferred by the very long, 788–1046 nt-long 3’ NCRs observed in other APV isolates remains to be explained.

With the extensive metagenomic analyses performed here, it becomes possible to hypothesize the origin of the cross-reactivity with PPV-specific reagents, observed in the *Prunus* sources analyzed here. The comparison of the virome of each *Prunus* source shows that APV2 is the only virus shared by all sources, with the exception of the Nanjing one, in which PPV-cross reactivity is directly explained by PPV infection. In addition, a similar analysis of two additional PPV cross-reacting sources (Agua and Ting Ting) [1, 2, 4] provided evidence for the presence of APV2 in co-infection with CGRMV and *Peach mosaic virus* (Agua source) or with PBNSPaV (Ting Ting source) (data not shown). However, since the APV2 genome coverage was limited in these analyses, no further efforts were made to characterize more precisely the APV2 isolates involved. Taken together, these results would seem to exclude a contribution of APV1 and APV3 to the serological cross reactions with PPV but make APV2 the likely candidate involved in cross-reactivity, in particular when considering that it is the only viral agent that was detected in the Bonsai source. Further investigations are clearly necessary to experimentally validate this hypothesis.

Questions also persist concerning the biology and pathogenicity of APV in *Prunus* materials. Unlike for other foveaviruses [21], no APV vector is known. APV are graft-transmissible, and dispersal likely occurs through infected propagation material, raising the question of their prevalence in such *Prunus* materials. The potential contribution of APV to the symptoms observed in the *Prunus* sources in which they were detected is also difficult to address. For one thing, these symptoms were very diverse: enlargement and discoloration of veins on old leaves, chlorotic leaf-spotting, fruit deformation and size reduction, delayed maturation [34]. In parallel, most of the sources showed complex mixed infections with a range of other fruit tree-infecting viruses. The situation is a bit different in the case of the Bonsai source, in which a single APV2 infection was detected. The original *P*. *mume* plant, grown as a bonsai, did not display any specific symptoms (J.B. Quiot, personal communication). However, GF305 peach seedlings grafted with that source showed enlarged veins on old leaves, a symptom also observed in GF305 indicators grafted with some of the other sources [34]. Although far from providing a conclusive link between APV and symptomatology, this observation suggests that at least APV2 could contribute to symptoms in at least the GF305 peach indicator. Again, further studies are necessary to determine potential pathogenicity of the various APV on different *Prunus* hosts.

## Supporting Information

S1 Fig Multiple alignment of the 3' NCR sequences from Asian prunus virus 3 isolates Ta Tao 23 (two variants), Nanjing and Ta Tao 25. (PDF)

S1 Table List of primers used to amplify and sequence internal gaps, and terminal regions for Asian prunus virus isolates identified in the Bungo, Bonsai, Nanjing, Ta Tao 23 and Ta Tao 25 *Prunus* sources. (DOCX)

## References

[1] Hadidi A, Levy L. Accurate identification of *Plum pox potyvirus* and its differentiation from Asian prunus latent potyvirus in *Prunus germplasm*. 1994; EPPO Bull. 24: 633–643

[2] James D, Thompson DA, Godkin SE. Cross reactions of an antiserum to *Plum pox potyvirus*. 1994; EPPO Bull. 24: 605–614

[3] Hari V, Abdel-Ghaffar MH, Levy L, Hadidi A. Asian prunus latent virus: an unusual Potyvirus detected in germplasm from east asia. 1995; Acta Hortic. 386: 78–85

[4] James D, Godkin SE, Eastwell KC, MacKenzie DJ. Identification and differentiation of Prunus virus isolates that cross-react with *Plum pox virus* and *Apple stem pitting virus* antisera. 1996; Plant Dis. 80: 536–543.

[5] Marais A, Svanella-Dumas L, Foissac X, Gentit P, Candresse T. Asian prunus viruses: new related members of the family *Flexiviridae in Prunus* germplasm of Asian origin. 2006; Virus Res. 120: 176–183. 16621102 10.1016/j.virusres.2006.03.004

[6] Marais A, Svanella-Dumas L, Foissac X, Candresse T. Molecular characterization of a new Foveavirus in *Prunus* accessions of Asian origin. 2004; Acta Hortic. 657: 87–92.

[7] Marini DB, Gibson PG, Scott SW. The complete nucleotide sequence of an isolate of Asian prunus virus 1 from peach [*Prunus persica* (L) Batch]. 2009; Arch Virol. 154: 1375–1377. 10.1007/s00705-009-0443-4 19578926

[8] Gibson P, Reighard G, Scott S, Zimmerman M. Identification of graft-transmissible agents from Ta Tao 5 peach and their effects on Coronet peach. 2001; Acta Hort 500: 309–314.

[9] Foissac X, Svanella-Dumas L, Gentit P, Dulucq MJ, Marais A, Candresse T. Polyvalent degenerate oligonucleotides reverse transcription-polymerase chain reaction: a polyvalent detection and characterization tool for Trichoviruses, Capilloviruses, and Foveaviruses. Phytopathology 2005; 95: 617–625. 10.1094/PHYTO-95-0617 18943777

[10] Massart S, Olmos A, Jijakli H, Candresse T. Current impact and future directions of high throughput sequencing in plant virus diagnostics. Virus Res. 2014; 188: 90–96. 10.1016/j.virusres.2014.03.029 24717426

[11] Boonham N, Kreuze J, Winter S, van der Vlugt R, Bergervoet J, Tomlinson J, et al Methods in virus diagnostics: From ELISA to next generationn sequencing. 2014; Virus Res. 186: 20–31. 10.1016/j.virusres.2013.12.007 24361981

[12] Barba M, Czosnek H, Hadidi A. Historical perspective, development and applications of Next-Generation Sequencing in plant virology. Viruses 2014; 6: 106–136. 10.3390/v6010106 24399207 PMC3917434

[13] Wu Q, Ding SW, Zhang Y, Zhu S. Identification of viruses and viroids by next-generation sequencing and homology dependent and homology independent algorithms. Annu Rev Phytopathol. 2015; 53: 425–444. 10.1146/annurev-phyto-080614-120030 26047558

[14] Marais A, Faure C, Couture C, Bergey B, Gentit P, Candresse T. Characterization by deep sequencing of divergent *Plum bark necrosis stem pitting-associated virus* isolates and development of a broad-spectrum PBNSPaV detection assay. Phytopathology 2014; 104: 660–666. 10.1094/PHYTO-08-13-0229-R 24328491

[15] Gentit P, Foissac X, Svanella-Dumas L, Peypelut M, Candresse T. Characterization of two different apricot latent virus variants associated with peach asteroid spot and peach sooty ringspot diseases. 2001; Arch Virol. 146: 1453–1464. 11676410 10.1007/s007050170071

[16] Candresse T, Marais A, Faure C, Gentit P. Association of *Little cherry virus 1* with the Shirofugen stunt disease and characterization of the genome of a divergent LChV1 isolate. Phytopathology 2013; 103: 293–298. 10.1094/PHYTO-10-12-0275-R 23402630

[17] Marais A, Svanella-Dumas L, Barone M, Gentit P, Faure C, Charlot G. et al Development of a polyvalent RT-PCR detection assay covering the genetic diversity of Cherry capillovirus A (CVA). Plant Pathol. 2011; 61: 195–204.

[18] Youssef F, Marais A, Faure C, Barone M, Gentit P, Candresse T. Characterization of *Prunus*-infecting *Apricot latent virus*-like Foveaviruses: Evolutionary and taxonomic implications. Virus Res. 2011; 155: 440–445. 10.1016/j.virusres.2010.11.013 21144869

[19] Tamura K, Stecher G, Peterson D, Filipski A, Kumar S. MEGA6: Molecular Evolutionary Genetics Analysis version 6.0. Mol Biol Evol. 2013; 3: 2725–2729. 10.1093/molbev/mst197 PMC3840312 24132122

[20] Martin DP, Murrell B, Golden M, Khoosal A, Muhire B. RDP4: Detection and analysis of recombination patterns in virus genomes. 2015; Virus Evol. 1: vev003 10.1093/ve/vev003 27774277 PMC5014473

[21] Adams MJ, Candresse T, Hammond J, Kreuze JF, Martelli GP, Namba S et al Family *Betaflexiviridae* In: King AMQ, Adams MJ, Carstens EB, Lefkowitz EJ, editors. Virus Taxonomy—Ninth Report on the International Committee on Taxonomy of Viruses. Elsevier Academic Press; 2012 pp 920–941.

[22] Abou Ghanem-Sabanadzovic N, Tzanetakis IE, Sabanadzovic S. Rubus canadensis virus 1, a novel betaflexivirus identified in blackberry. Arch Virol. 2013; 158: 445–449. 10.1007/s00705-012-1484-7 23053515

[23] James D, Varga A, Jesperson GD, Navratil M, Safarova D, Constable F et al Identification and complete genome analysis of a virus variant or putative new foveavirus associated with apple green crinkle disease. Arch Virol. 2013; 158: 1877–1887. 10.1007/s00705-013-1678-7 23553453

[24] James D, Varga A, Lye D. Analysis of the complete genome of a virus associated with twisted leaf disease of cherry reveals evidence of a close relationship to unassigned viruses in the family *Betaflexiviridae*. Arch Virol. 2014; 159: 2463–2468. 10.1007/s00705-014-2075-6 24737006

[25] Szutba-Solinska J, Urbanowicz A, Figlerowicz M, Bujarki JJ. RNA-RNA recombination in plant virus replication and evolution. Annu Rev Phytopathol. 2011; 49: 415–443. 10.1146/annurev-phyto-072910-095351 21529157

[26] Martelli GP, Adams MJ, Kreuze JF, Dolja VV. Family *Flexiviridae*: A case study in virion and genome plasticity. Annu Rev Phytopathol. 2007; 45: 73–100. 17362202 10.1146/annurev.phyto.45.062806.094401

[27] Singh L, Hallan V, Martin DP, Ram R, Zaidi AA. Genomic sequence analysis of four new *Chrysanthemum virus B* isolates: evidence of RNA recombination. Arch Virol. 2012; 157: 531–537. 10.1007/s00705-011-1190-x 22179900

[28] Marais A, Faure C, Mustafayev E, Candresse T. Characterization of new isolates of *Apricot vein clearing-associated virus* and of a New *Prunus*-Infecting Virus: Evidence for recombination as a driving force in *Betaflexiviridae* evolution. PLoS One. 2015; 10(6):e0129469 10.1371/journal.pone.0129469 26086395 PMC4472227

[29] Zanardo LG, Silva FN, Lima ATM, Milanesi DF, Castilho-Urquiza GP, Almeida AMR et al Molecular variability of *Cowpea mild mottle virus* infecting soybean in Brazil. Arch Virol. 2014; 159: 727–737. 10.1007/s00705-013-1879-0 24142270

[30] Alabi OJ, Al Rwahnih M, Mekuria TA, Naidu RA. Genetic Diversity of *Grapevine virus A* in Washington and California Vineyards. Phytopathology 2014; 104: 548–560. 10.1094/PHYTO-06-13-0179-R 24168043

[31] Villamor DEV, Eastwell KC. Viruses associated with rusty mottle and twisted leaf diseases of sweet cherry are distinct species. Phytopathology 2013; 103: 1287–1295. 10.1094/PHYTO-05-13-0140-R 24219146

[32] Yoon JY, Joa JH, Choi KS, Do KS, Lim HC, Chung BM. Genetic diversity of a natural population of Apple stem pitting virus isolated from apple in Korea. Plant Pathol J. 2014; 30: 195–199. 10.5423/ 10.5423/PPJ.NT.02.2014.0015 25289003 PMC4174845

[33] Simmonds J. Methods for virus classification and the challenge of incorporating metagenomic sequence data. 2015; J Gen Virol. 96: 1193–1206. 10.1099/vir.0.000016 26068186

[34] Desvignes JC, Boye R, Cornaggia D, Grasseau N. Virus Diseases of Fruit Trees. 1999; Ed Ctifl, France.

