# Supplementary material for: New Insights into Asian Prunus Viruses in the Light of NGS-Based Full Genome Sequencing
Source: PLoS One. 2016 Jan 7;11(1):e0146420. doi: 10.1371/journal.pone.0146420 (PMC4704818; doi:10.1371/journal.pone.0146420)
Supplement: S1 Fig — (PDF) [file pone.0146420.s001.pdf]

```
                    1                                                                                                    100
      APV3_Nanjing  TAGTCACCCA GTCACCAGAG GTTGAATATA AATGGTGATT AGCGTTCCCG TAGCCGTTGA GGGTGAAGCA ACGGAGCCTC AATTCTATCT CTAAATGGGC
         APV3_TT25  TAGCCACCAG ATTGCAAGAA GTTGTGCATG AAGGGTAGTT .......... .......... .......... .......... .......... ..........
APV3_TT23_variant1  TAGCCACCAG ATTGCCTGGA GTTG...... .......... .......... .......... .......... .......... .......... ..........
APV3_TT23_variant2  TAGCCACCAG ATTGCCTGGA GTTGT..... .......... .......... .......... .......... .......... .......... ..........

                    101                                                                                                  200
      APV3_Nanjing  TTTAAAGAAG CTTAAAGTGT CAATGGTGCA CCTCGGCAAG AGGTTTCCAG ACAAGCTTTT AGTGACAGAA GACAGGTACT TGTCATCGTC CAGATGATGA
         APV3_TT25  .......... .......... .......... .......... .......... .......... .......... .......... .......... ..........
APV3_TT23_variant1  .......... .......... .......... .......... .......... .......... .......... .......... .......... ..........
APV3_TT23_variant2  .......... .......... .......... .......... .......... .......... .......... .......... .......... ..........

                    201                                                                                                  300
      APV3_Nanjing  GGATGGTTTA AATCGAGTCT TGGTGATCTG ACTATAAAAG AGCCAATTGG CCTAGAACCG TGGAGAGGCC TAAAAGAGTC CACGTGTCAT CTTAGAGCCA
         APV3_TT25  .......... .......... .......... .......... ......TTGG CCTAGAACCG TGGAGAGGCC TAAAAGAGTC CACGTGCCGT CTTAGAGTCA
APV3_TT23_variant1  .......... .......... ....TGCATG AAGGGTAGTT TGTGTTTTGG CCTAGAACCG TGGAGAGGCC TAAAAGAGTC CACGTGCCGT CTTAGAGTCA
APV3_TT23_variant2  .......... .......... .......... .......... .......... .......... .......... .......... .......... ..........

                    301                                                                                                  400
      APV3_Nanjing  TTAGGTGTGT TTTAGTAAAT ATTAGTAGTT TGCACTTCCC TGAGTTCCAG GTCTAGAACC CAGGAAAGAC CCAAAAGAGT CCTGGTGGGC GTTTAGCCAT
         APV3_TT25  TAGTATGTGT CCTAGTAAAT ATTTGTAGTT TCCACTTCCT TAGGTTCCAG GTCTAGAACC CAGGAGAGAC CCAAAAGAGT CCTGGTTGGC GCTTAGCCAC
APV3_TT23_variant1  TAGTATGTGT CCTAGTAGAT ATTTGTAGTT TCCACTTCCT TAGGTTCCAG GTCTA..... .......... .......... .......... ..........
APV3_TT23_variant2  .......... .......... .......... .......... .......... .......... .......... .......... .......... ..........

                    401                                                                                                  500
      APV3_Nanjing  T......... .......... .......... .... TTAAAAA CCTCTGCACC TCGTAGGCCA CACCAACGTC AATCATCGTG TGCCTTATGT GGGCAAGTTA
         APV3_TT25  AAAGAAGAGA AAAAGAAAAA AATTAAAAGG AAAAGAAAAA CCTCTGCATC GTATAGGCCA CACCAACGCT AATTATAGTG TGCCCTATGC GGGCAAGTTA
APV3_TT23_variant1  .......... .......... .......... .......... .......... .......... .......... .......... .......... ..........
APV3_TT23_variant2  .......... .......... .......... .......... .......... .......... .......... .......... .......... ..........

                    501                                                                                                  600
      APV3_Nanjing  ACCGTGTTGT GGCGGCAACC GGAAACGTTA GATGCAAGCG TAGATTCATG AGCTACGTCC TGTGGTTGGA TTTCCACCGT TATCTCGTCG TTAAGAGAGT
         APV3_TT25  ACCAGGTTGT GGCGGCAACC TGGAATGTCA GATGCAAGCG TAGATTTATG AGCTACGCCC TGTGGTTGGA TTTCCACCGT TATCTCATCG TCAAGAGAGC
APV3_TT23_variant1  .......... .......... .......... .......... .......... .......... .......... .......... .......... ..........
APV3_TT23_variant2  .......... .......... .......... .......... .......... .......... .......... .......... .......... ..........

                    601                                                                                                  700
      APV3_Nanjing  AACCCCTCTT CTCTCGCTTA TGGGGGCATC GTTAAGCGGG CGTGTGACCT ATTGAAAGGT GGGTTCCCTA CGTTAAGGGT AGTTGTGGTT TAGAATAAAC
         APV3_TT25  AACCCCTCTT TTCTCGCTTA TGGGGGTATT GTTAGGCGAG CGTGTGATCC ATTGAAAGAT GGGTTCCCTG CGTTAAGGGT AGTTGTGGTT TAGAACAAAC
APV3_TT23_variant1  .......... .......... .......... .......... .......... .......... .......... .......... .......... ..........
APV3_TT23_variant2  .......... .......... .......... .......... .......... .......... .......... .......... .......... ..........

                    701                                                                                                  800
      APV3_Nanjing  CTCCATGGTT CCGACCTGCC ATGTTTCAAG CGATCGGCAG TTCTGGGTAA GAAACAACCC TGTGCTTGGT GGTTAAGCAT GACCACTCAG TTTGACCAAC
         APV3_TT25  CTCCATGGTT CCGACCTACC ATGATTAAAG TGATCGGTAG TTCTGGGTAA GAAACAACCC TGTGCTTGGT GGCTAAGCAT GACCACTCAG TTTGACCGAC
APV3_TT23_variant1  .......... .......... .......... .......... .......... .......... .......... .......... .......... ..........
APV3_TT23_variant2  .......... .......... .......... .......... ........... ......... .......... .......... .......... ..........

                    801                                                                                                  900
      APV3_Nanjing  TGGACAGACA GGTTACGTTT CCCATTTCGG TATCAAAAGT GGGCGCATAT CAAAGCGTTA AATTGCTCAC CTGCCAGTTT TAAACCTTCT TTCCTG..TG
         APV3_TT25  TGGCCAGACA GGTTACGTTT CCCACTTCGA TATCAAAAGT GGGCGCATAT CAAAGCGTTA AATTGTTCAC TCGCGTGTCT CAAACTTCCA TTCTTGGGTG
```

```
APV3_TT23_variant1  .......... .......... .......... .......... .......... .......... .......... .......... .......... ..........
APV3_TT23_variant2  .......... .......... .......... .......... ........... ......... .......... .......... .......... TTCTTGGGTG

                    901                                                                                                   1000
      APV3_Nanjing  GTTTGGAACA GGCTTCAGTC CTGGTGATCT GACTTTAAAA GAGCCAAATA AACCGCATTT CAACGCGTTA TAGTGTTCGC TGACAGACCG TACAAATAGT
         APV3_TT25  GTTTGAAATG GGCCTTGGTC CTGGTGATCT GACCTTAAAA GAGCCAAATA AACCGCATTT CAACGCGTTA TAGTGTTCGC TGACAGACCG TACGAATAGT
APV3_TT23_variant1  .......... .......... ..GGTGATCT GACCTTAAAA GAGCCAAATA AACCGCATTT CAACGCGTTA TAGTGTTCGC TGACAGACCG TACGAGTAGT
APV3_TT23_variant2  GTTTGAAATA GGCCTTGGTC CTGGTGATCT GACCTTAAAA GAGCCAAATA AACCGCATTT CAACGCGTTA TAGTGTTCGC TGACAGACCG TACGAGTAGT

                    1001                                                                       1080
      APV3_Nanjing  CTGTTTCACC GACCTAAGAC CTAAGTAATT GAATAAGGGA AGGTGATTAA ACAAATTTGC TTTTTATCGC TTATTTTCCC
         APV3_TT25  CTGTTTCACC AACCTAAGAC TTAAATAATT GAATAAGGGG AGGTGATTAA ACAAGTTTGC TTTTTATCGC TAATTTTGC.
APV3_TT23_variant1  CTGTTTCACC AACCTAAGAC CTAAATAATT GAATAAGGGG AGGTGATTAA ACAAATTTGC TTTTTATCGC TAATTTTCC.
APV3_TT23_variant2  CTGTTTCACC AACCTAAGAC CTAAATAATT GAATAAGGGG AGGTGATTAA ACAAATTTGC TTTTTATCGC TAATTTTGT.
```
