# Supplementary material for: New Insights into Asian Prunus Viruses in the Light of NGS-Based Full Genome Sequencing
Source: PLoS One. 2016 Jan 7;11(1):e0146420. doi: 10.1371/journal.pone.0146420 (PMC4704818; doi:10.1371/journal.pone.0146420)
Supplement: S1 Table — (DOCX) [file pone.0146420.s002.docx]

**S1 Table. List of primers used to amplify and sequence internal gaps, and terminal regions for Asian prunus viruses identified in Bungo, Bonsai, Nanjing, Tatao 23 and Tatao 25 *Prunus* sources.**

|  | Sequence 5'-3' | Lenght of PCR product (bp) |
| --- | --- | --- |
| **APV1 Bungo** |  |  |
| Race-APV1-Bun^a^ | GGATTGAGGTCAGCCCCGAAAATGAAGCTT | 1704 |
| APV1-Bun-F1 | GTTCTTCCATCAATGGAGCTCGCCC | 355 |
| APV1-Bun-R1 | ATGTGACATGTGGCGTTGAC |  |
| APV1-Bun-F2 | ATGATCATCCCAACGGAGCA | 1176 |
| APV1-Bun-R2 | TAATGAAACGCACGCAGCTA |  |
| APV1-Bun-F3 | CTTTGACTTGTTAGACAGGC | 477 |
| APV1-Bun-R3 | ATACATGGATGGCAGTAACC |  |
| APV1-Bun-F4 | TCACTTGAAAGTTGGAAAG | 440 |
| APV1-Bun-R4 | CAAAAGTTATGTCCTTTGTG |  |
| LD-APV1-Bun^b^ | CAATCGAGTGATGAAATCTGCTGTTA | 877 |
|  |  |  |
| **APV2 Bungo** |  |  |
| Race-APV2-Bun^a^ | CGGGAGGAAAGACCACCGTTGCTACC | 690 |
| APV2-Bun-F1 | GACCCTCAAGAAGCCTGACTTG | 596 |
| APV2-Bun-R1 | ACCTCCTTTGGAACACATTG |  |
| LD-APV2-Bun^b^ | GAGCTACGCCCTGTGGTTGGAT | 530 |
|  |  |  |
| **APV2 Bonsai** |  |  |
| Race-APV2-Bon^a^ | AGCTGAGGGGTTGTGTGTATGACTCGC | 813 |
| APV2-Bon-F1 | CTTCTCATGCCAGACACCGC | 378 |
| APV2-Bon-R1 | CACTCTTCTAACCAACCTCTT |  |
| LD-APV2-Bon^b^ | GACAGGCTTTAGTCCTGGTGATCT | 153 |
|  |  |  |
| **APV2 Ta Tao 25** |  |  |
| LD-APV2-TT25^b^ | TTTAGCTGTATAAAACCTCTGCAC | 690 |
|  |  |  |
| **APV3 Nanjing** |  |  |
| Race-APV3-Nan^a^ | AGCCCCAGCGAGTATCTCTGGTGGGA | 707 |
| APV3-Nan-F1 | GCTCAGTCAAATCATTCCTAA | 600 |
| APV3-Nan-R1 | GGGTTAAGATCGGCTCCGAAG |  |
| APV3-Nan-F2 | CATAGAGGTAGTGACTCCGT | 363 |
| APV3-Nan-R2 | AAAACAAGCCAGGGTCTGCC |  |
| LD-APV3-Nan^b^ | AGTGGGCGCATATCAAAGCG | 241 |
|  |  |  |
| **APV3 Ta Tao 25** |  |  |
| APV3-TT25-F1 | ACTCTACAGGTACATTCGGAC | 489 |
| APV3-TT25-R1 | CCATCAAGAACATGGTGCAGA |  |
| APV3-TT25-F2 | TGTGTCTCCAAGGAGGTCT | 404 |
| APV3-TT25-R2 | CCTTGGTATCCATCCTGCAA |  |
| LD-APV3-TT25^b^ | GGTGTCATCTTGCACCGGACCG | 999 |
|  |  |  |
| **APV3 Ta Tao 23** |  |  |
| APV3-TT23-F1 | TCTGTCCAAGCGGCCAAGTC | 794 |
| APV3-TT23-R1 | AGCCACAGAAGATACATAACTAC |  |
| APV3-TT23-F2 | GAGCCAGCTCTTCCAGTGAA | 815 |
| APV3-TT23-R2 | AGACAGAGCATCATTAAGTCC |  |
| APV3-TT23-F3 | CCTGACGACCACCCGAACA | 333 |
| APV3-TT23-R3 | CCTACATGAGCGTCCAGAAT |  |
| LD-APV3-TT23^b^ | GGTGTCATCCTGCACCGAACCG | 432 (variant 1) |
|  |  | 334 (variant 2) |

^a^ This reverse primer was used in conjunction with the universal primer provided by the 5’ RACE kit (Takara Bio Europe/Clontech).

^b^ In conjunction with LD prime primer (5' CACTGGCGGCCGCTCGAGCATGTAC 3')
